# Sky Image Classification Based on Transfer Learning Approaches

**DOI:** 10.3390/s24123726

**Published:** 2024-06-08

**Authors:** Ruymán Hernández-López, Carlos M. Travieso-González, Nabil I. Ajali-Hernández

**Affiliations:** Signals and Communications Department (DSC), Institute for Technological Development and Innovation in Communications (IDeTIC), University of Las Palmas de Gran Canaria (ULPGC), 35017 Las Palmas de Gran Canaria, Spain; ruyman.hernandez@ulpgc.es (R.H.-L.); nabil.ajali101@alu.ulpgc.es (N.I.A.-H.)

**Keywords:** cloudiness classification, deep learning, transfer learning, convolutional neural networks, EfficientNet models, ResNet models, sky images, photovoltaic power, renewable energy

## Abstract

Cloudy conditions at a local scale pose a significant challenge for forecasting renewable energy generation through photovoltaic panels. Consequently, having real-time knowledge of sky conditions becomes highly valuable. This information could inform decision-making processes in system operations, such as determining whether conditions are favorable for activating a standalone system requiring a minimum level of radiation or whether sky conditions might lead to higher energy consumption than generation during adverse cloudy conditions. This research leveraged convolutional neural networks (CNNs) and transfer learning (TL) classification techniques, testing various architectures from the *EfficientNet* family and two *ResNet* models for classifying sky images. Cross-validation methods were applied across different experiments, where the most favorable outcome was achieved with the *EfficientNetV2-B1* and *EfficientNetV2-B2* models boasting a mean *Accuracy* of 98.09%. This study underscores the efficacy of the architectures employed for sky image classification, while also highlighting the models yielding the best results.

## 1. Introduction

The production of electric power from solar energy is influenced by variations caused by solar obstruction due to cloud cover. Detecting and understanding cloud cover have been investigated for estimating and forecasting solar irradiance, and in this way, predicting photovoltaic power generation [1]. The amount of electrical energy generated in a photovoltaic plant is not solely determined by infrastructure specifics like the number and type of solar panels employed, it is also directly impacted by the radiation received by the panels, hence, the prevailing cloud cover. Sky conditions are contingent upon the time of day, season, and geographical location. Nevertheless, forecasting local cloud conditions remains highly challenging.

Furthermore, identifying specific cloud cover conditions through automatic pattern recognition in sky images can prove invaluable across various applications. For instance, it could aid in automated decision-making processes governing the operation of systems in remote, off-grid locations where power consumption is critical. In such scenarios, it becomes crucial to ascertain whether conditions are conducive for activating a standalone system requiring a minimum radiation threshold or if cloudier conditions might result in higher consumption than energy generation, potentially leading to system failure.

### 1.1. Related Work

In contemporary times, a plethora of research endeavors are dedicated to gauging solar irradiation levels and assessing sky conditions via image processing. In recent years, several surveys have scrutinized cloud conditions utilizing various pattern recognition technologies and diverse approaches to image acquisition.

For instance, a study conducted in 2015 by Alonso-Montesinos et al. [2] utilized a sky camera to forecast short-term and medium-term solar radiation for various sky conditions (clear, partly cloudy, and cloudy skies) at one-minute intervals. This research explored the potential of the camera as a ground-based predictive tool solely relying on digital image levels. The radiation forecast was derived through a pixel-level radiation estimation method and Cloud Motion Vectors (CMV), enabling the calculation of motion for each pixel within the images.

However, in recent years, the techniques commonly employed have shifted towards deep learning (DL) technologies due to their promising results and the increasing prevalence of this type of technology, which offers significant improvements in pattern recognition.

During this decade, numerous studies focusing on analyzing sky images using deep learning (DL) techniques have emerged. One such study was conducted by Seongha Park et al. [3], where they illustrate the potential of sky-facing cameras coupled with machine learning (ML) methods to estimate solar power output. This research specifically proposes cloud segmentation to determine cloudiness for estimating photovoltaic production. The authors compare various methodologies, including a classical regression model, DL techniques, and boosting methods that integrate results from other ML models. For cloudiness estimation, three types of Deep Neural Network (DNN) architectures were employed: Fully Convolutional Network (FCN), U-shaped Network (U-Net), and DeepLabv3. Notably, the most accurate segmentation of cloud pixels was obtained with one of the DNNs: the U-Net architecture.

In the same year (2021), two months later, another study was published [4], conducted by Rial A. Rajagukguk et al. In this study, the Long Short-Term Memory (LSTM) algorithm was utilized to distinguish between various sky conditions, including clear sky, overcast sky, and partly cloudy sky. This distinction aimed to predict fluctuations in solar radiation and, consequently, in solar energy output within a photovoltaic plant.

The studies referenced in the preceding paragraphs undertake pixel-level cloud detection to forecast solar radiation. However, despite the existence of techniques to address the fuzzy edges, diverse shapes, and textures of clouds [5], deep learning (DL) methods can also be employed to predict net radiation.

Enrique Nueve et al. proposed a deep learning approach for nowcasting net radiation within subhourly and intrahour horizons, as outlined in their study [6]. This method aims to enhance the understanding and management of processes influenced by net radiation, which holds critical importance for renewable energy planning and agriculture. The WeatherNet model they developed is based on the deep-learning architecture CNN-LSTM (ConvLSTM), which integrates data from multiple local ground-based cameras and weather sensors to predict net radiation. Notably, their approach differs from previous methodologies by incorporating imagery from three distinct cameras: a sky-facing RGB (Red Green Blue) camera, a horizon-facing RGB camera, and a horizon-facing forward-looking infrared camera. Additionally, they employ a Gated Recurrent Unit (GRU), a type of Recurrent Neural Network (RNN) architecture similar to LSTM, in their work.

In a separate study by Quentin Paletta et al. [7], four commonly utilized deep learning architectures were compared in their ability to forecast solar irradiance based on sequences of hemispherical sky images and exogenous variables: a CNN model, a CNN + LSTM model, a 3D convolutional neural network (3D-CNN), and a ConvLSTM. As a critical facet of data-driven approaches, the selection of the dataset significantly influences the performance of DL models. The research suggests that training the model with a sufficient volume of data or aggregated datasets from multiple locations may enhance the learning of the model as much, if not more than, relying solely on architectural modifications for these types of learning technologies.

In their study [8], Youssef Karout et al. introduced a hybrid model integrating DNI (Direct Normal Irradiance) measurements with sky-imaging data. This research presents a model specifically designed for intrahour forecasting of DNI, with horizons spanning 5, 10, and 15 min. The hybrid model combines a knowledge-based approach with a machine learning model: the knowledge-based component forecasts clear-sky DNI based on DNI measurements, while the machine learning component assesses the impact of atmospheric disturbances on solar resources by analyzing high dynamic range sky images captured by ground-based cameras. In this instance, Recurrent Neural Networks (RNNs) were employed, including LSTM and a CNN-LSTM network.

On the other hand, Gyasi and Swarnalatha proposed an architecture based on MobileNet in their study [9]. Their model, Cloud-MobiNet, is a lightweight architecture designed for implementation on smartphones. It comprises two components: the MobileNet building block and the support MobileNet block. This model achieved an overall accuracy of 97.45% and is considered valuable not only for meteorological analysis and forecasting but also for applications in the aeronautical and aviation industries.

As observed, this type of technology is not only beneficial in electric power production. As a further field of application, it is also crucial for precision agriculture applications. Sky conditions, particularly cloud shadowing, play a pivotal role in determining the quality of images captured by low-altitude sensing platforms. For instance, in a study by Czarnecki et al. [10], various deep learning approaches were compared to classify sky conditions, particularly regarding cloud shadows in agricultural fields, using visible spectrum cameras. Subsequently, they developed an artificial intelligence-based edge computing system to automate the classification process entirely. The training dataset comprised 100 oblique angle images of the sky, which were fed into a CNN and two deep residual neural networks. Both pre-trained and non-pre-trained versions of ResNet18 and ResNet34 were employed, with the pre-trained models utilizing the ImageNet database. Their findings revealed that ResNet18 and ResNet34 classifiers outperformed a traditional CNN classifier in terms of classification accuracy. The highest overall accuracy of 92% was achieved by ResNet34. Furthermore, the study utilized up to 13 cameras, albeit they were trail cameras.

Similar to the preceding study, the transfer learning technique was also employed in another investigation [11], this time utilizing Total Sky-Imager (TSI) images. In this scenario, the chosen architectures consisted of the AlexNet and ResNet101 models, which were subsequently trained using an Ensemble Learning approach to model and predict solar radiation.

Similarly to the study conducted by Gyasi and Swarnalatha, Guzel et al. developed models capable of distinguishing among ten classes of cloud conditions, in addition to one class dedicated to airplane vapor trails. Their research [12] utilized architectures based on MobileNet v2, VGG-16, ResNetV2-152, InceptionV3, EfficientNetV2-L, and ConvNeXtSmall. However, they surpassed the best result achieved by Gyasi and Swarnalatha, obtaining an overall accuracy of 97.66% by employing the transfer learning technique in a model based on the Xception architecture.

Indeed, all the studies presented thus far make use of images taken from Earth. However, it is noteworthy that images from Earth-observing satellites have predominantly been utilized to analyze cloud types and solar irradiance over large areas. For instance, multispectral imagers have been deployed for surface imaging across multiple wavelengths. The intensity of reflection from each wavelength is utilized to distinguish different cloud types [13,14]. Consequently, satellites have primarily been employed to detect multilevel clouds in large areas, using techniques such as the subregioning of clouds [15] and superpixel methods [16] to enhance cloud detection accuracy.

Nevertheless, ground-based sky-facing cameras are better suited for the estimation and prediction of solar irradiance and weather conditions on a local scale [3]. Moreover, in the domain of solar energy, short-term changes in electricity production caused by occluding clouds can be predicted at different time scales. All-sky cameras enable predictions up to 30 min ahead, while satellite observations extend predictions up to 6 h ahead [17].

### 1.2. Contributions

The research outlined in this article has been undertaken with the aim of automatically classifying sky images according to various sky conditions, including clear, partly cloudy, and cloudy skies.

**Figure 1:**
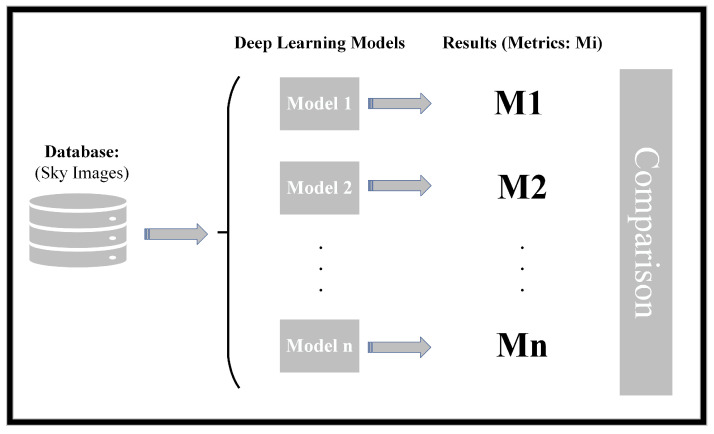
Conceptual schematic representation of the work carried out.

The conceptual schematic diagram of the work carried out is given in Figure 1.

As outlined, the first step involved creating a database comprising images depicting various sky conditions for the study. During this process, the samples were labeled according to different types of cloud cover. Once the database was established, various classification models were implemented. Subsequently, the samples were fed into these models for classification. The classification results were then assessed using different metrics to evaluate the performance of each implemented model. Finally, a comparison of the different models utilized in this study was conducted, based on the outcomes reflected by these model evaluation metrics.

The novelty of this study, compared to the current state of the art, lies in its comprehensive testing of all *EfficientNet* architectures available in the *Keras* library [18] at the time of writing, for the classification of sky images. The aim was to classify sky images and detect the type of cloudiness present in each sample. While there are studies involving various machine learning techniques to predict cloud cover in sky images, some of which utilize *EfficientNet* models, such as [19] focusing on a specific model (*EfficientNet-B0*), none have been found that apply *EfficientNet* models as extensively, including version 2 models [20], to discriminate among these type of images.

Nonetheless, this study also delved into experimenting with the residual network architectures *ResNet-50* and *ResNet-101* [21]. The rationale behind utilizing solely these two architectures in this study lies in their superior performance observed in prior experiments where other residual networks were tested. In these prior studies, sky image classification was conducted, employing the following architectures: *ResNet-50*, *ResNet-50V2*, *ResNet-101*, *ResNet-101V2*, *ResNet-152*, and *ResNet-152V2*. But, it must be taken into account that *ResNetV2* and the original ResNet (version 1) vary primarily in that version 2 applies batch normalization before each weight layer.

Hence, the aim of this research was to assess the applicability of utilizing these *EfficientNet* and *ResNet* models for classifying cloudiness in sky images, with the objective of identifying the models that yield the most optimal outcomes.

This paper is organized as follows: First, the Materials and Methods section will present an overview of the materials used and the research methodology. Next, the Experimental Methodology section will elaborate on the experimental procedures and detail how the experiments were conducted. Subsequently, a dedicated section will present the results obtained from the experiments, providing an in-depth analysis and interpretation. Finally, the Conclusion section will offer insights and implications arising from the research findings.

## 2. Materials and Methods

This section delineates the dataset composition employed in the experiments of this study and its relevance to the research. Additionally, it provides an overview of the deep learning techniques and models implemented in the study.

### 2.1. Datasets and Data Selection

In the field of pattern recognition, having a suitable learning dataset is essential. From the original dataset, the training dataset is derived, and this set plays a central role because it is used to train, evaluate and, therefore, ultimately construct the classifier.

Currently, there are several platforms and public databases accessible for obtaining sky images. To name some of them, the following resources can be found:

- The waggle edge computing framework [22], which is an open sensor platform for edge computing;
- The datasets provided by the Site Instrumental de Recherche par Télédétection Atmosphérique (SIRTA) laboratory [23];
- The Singapore Whole-sky IMaging CATegories (SWIMCAT) database [24];
- Total Sky-Imager images of the Southern Great Plains (SGP) from the Atmospheric Radiation Measurement (ARM) dataset [25];
- The Hybrid Thresholding Algorithm (HYTA) database [26];
- The SKy Images and Photovoltaic Power Generation Dataset (SKIPP’D) [27]: a publicly available standardized benchmark dataset for image-based solar forecasting, containing three years (2017–2019) of quality-controlled down-sampled sky images and photovoltaic power generation data for short-term solar forecasting using deep learning.

Nonetheless, this study relies on a distinct dataset assembled specifically for this research. While sky conditions are influenced by various climatic variables such as rain or wind, as well as environmental factors like air pollution or seasonal changes, these are inherently tied to specific geographical locations, which significantly affect the potential for photovoltaic energy generation. The image dataset utilized in this study comprises sky images captured specifically from the southeast region of Gran Canaria Island (the Canary Islands, Spain).

The initial database comprises images captured using a Vivotek omnidirectional IP camera, specifically the FE8391-EHV model, recognized for its capability to provide high-resolution images (5 megapixels) and a 360° surround view facilitated by its fisheye lens. The imaging process was executed through a cost-effective, efficient, and reliable system, equipped with a backup subsystem that generates daily graphs depicting solar energy generation. This setup greatly aids in the detection of sky conditions. Furthermore, this photovoltaic system streamlines the selection and labeling of images required for subsequent sky image classification tasks. For a more comprehensive understanding of the image acquisition system, including details on data acquisition, analysis, and archiving procedures, further information is available in the related paper [28].

The sky images were captured every 2 s and organized into directories following the year/month/day sequence. Afterward, the samples were resized to 400×400 pixels and stored in new directories nested within the daily directories. As a result, the dataset comprises over 4 months of recordings, taken at two different resolutions, totaling over 5 million all-sky images.

To conduct the experiments in this study, samples were meticulously chosen to encompass various cloud conditions. The resulting dataset comprises images captured between 22 March 2021, and 13 September 2021. This meticulous selection process ensured the creation of a well-balanced dataset containing three distinct classes: clear sky, partly cloudy sky, and cloudy sky. Each class consists of a total of 1500 samples. Hence, this research employed a dataset comprising 4500 RGB images, encoded in PNG format, and resized to 400×400 pixels. A summary of the selected samples constituting the dataset is presented in Table 1.

**Figure 2:**
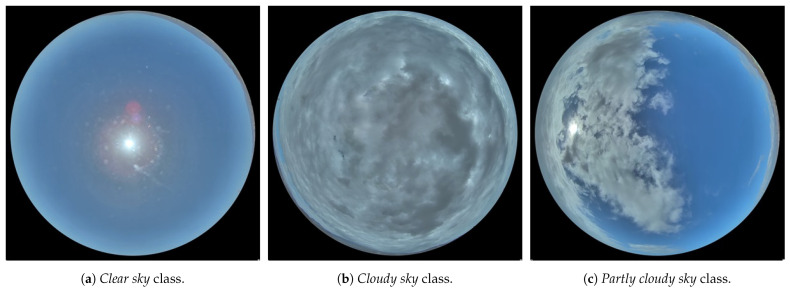
Representative samples of 400 × 400 pixels that make up the database of sky images selected for the experiments of this research. (**a**) Sky image labeled with the class *clear sky*. (**b**) Sky image labeled with the class *cloudy sky*. (**c**) Sky image labeled with the class *partly cloudy sky*.

Representative samples of each of these classes can be seen in Figure 2: Figure 2a shows an image with a completely clear sky, Figure 2b shows an image with a overcast sky, and Figure 2c shows an image with a partly cloudy sky.

While each sample in Figure 2 serves as a clear representation of its respective class, it is essential to note that not all dataset samples exhibit such distinct categorization. In some instances during labeling, uncertainty arose regarding whether specific samples should be classified as *clear sky* or *partly cloudy sky*. Similarly, uncertainty sometimes arose when determining whether a sample should be labeled as *partly cloudy sky* or *cloudy sky*. This ambiguity stems from the inability to establish a precisely defined boundary between these classes, consequently resulting in misclassification errors for such samples.

Nevertheless, the primary objective of this research was to assess the discriminative capabilities of the models under study concerning the various cloudiness conditions depicted in sky images. To achieve this goal, a diverse range of sky conditions was available across all three classes.

### 2.2. Recognition of Sky Conditions Using Deep Learning Approaches

The classification algorithms in this research were implemented using the machine learning platform *TensorFlow v2.15*. *TensorFlow* is an open-source platform that offers an end-to-end solution for machine learning tasks, providing a flexible ecosystem of tools, libraries, and community resources. It empowers researchers to advance the state of the art in ML, while enabling developers to easily create and deploy ML-powered applications [29]. Particularly, these classification models were developed using *Keras* [18], a deep learning API (*Application Programming Interface*) written in *Python* that operates on top of the machine learning platform *TensorFlow* [30].

*TensorFlow* serves as the infrastructure layer for differentiable programming, handling tensors, variables, and gradients. In contrast, *Keras* acts as a user-friendly interface for deep learning, managing layers, models, optimizers, loss functions, and metrics, among other functionalities. Essentially, *Keras* functions as the high-level API for *TensorFlow*. Moreover, *Keras* applications provide transfer learning models equipped with pre-trained weights. These models are versatile, facilitating tasks such as prediction, feature extraction, and fine-tuning.

Deep learning, particularly convolutional neural networks (CNNs), has significantly enhanced the learning capabilities of intelligent algorithms [31]. CNNs, a subclass of Artificial Neural Networks (ANNs), are primarily employed for image analysis and have the ability to learn directly from data. Utilizing convolutional layers, pooling layers, and fully connected layers, CNNs empower computational models to represent data with diverse levels of abstraction.

#### 2.2.1. Cloud Recognition with *ResNet* and *EfficienNet* Models

On one hand, there is evidence suggesting that the depth of the network is crucial [32,33], as deeper neural networks can enrich feature levels through stacked layers (depth). This raises the question of whether improving network learning is as straightforward as adding more layers.

However, addressing this question is hindered by the notorious problem of vanishing/exploding gradients, which obstruct convergence from the outset. When deeper networks are able to start converging, another issue arises: a degradation problem surfaces, whereby increasing network depth leads to the saturation of *Accuracy*, followed by rapid deterioration. This degradation, which is distinct from overfitting, is evident in higher training errors with the addition of more layers to appropriately deep models. The decline in training accuracy indicates that optimizing all systems may not be equally straightforward.

In addressing this optimization challenge, Kaiming He et al. introduced residual neural networks [21] to enhance plain networks by incorporating shortcut connections, effectively transforming them into their residual counterparts. Building on these insights, this study aimed to evaluate the performance of *ResNet* models in classifying sky images.

On the other hand, CNNs are often initially designed with a predetermined resource allocation and later scaled up to enhance *Accuracy* if more resources become accessible. The *EfficienNet* family of models [34] introduces a novel scaling approach that uniformly adjusts all dimensions of depth, width, and resolution using a straightforward yet highly effective *compound coefficient*.

Moreover, *EfficienNet* models have demonstrated superior performance compared to other architectures [20,34], achieving *Accuracy* values that sometimes surpass 90% on certain transfer learning datasets. Therefore, it was deemed worthwhile to assess their effectiveness in classifying sky images.

#### 2.2.2. Transfer Learning and Recognition Models

The deep learning technique underlying the classifiers implemented in this study is known as *transfer learning*. Many machine learning methods operate effectively only under a common assumption: that the training and test data are drawn from the same feature space and distribution. When the distribution changes, most statistical models must be rebuilt from scratch using newly collected training data. However, in many real-world scenarios, it is either expensive or impractical to collect the necessary training data and rebuild the models. Thus, reducing the need and effort to collect training data becomes essential. In such cases, knowledge transfer or transfer learning between task domains becomes desirable [35].

Transfer learning is a machine learning approach where a model developed for one task is reused as a starting point for a model in another task [36]. This is facilitated by the reuse of pre-trained weights, which involves utilizing neural networks that have been previously trained on certain data. Consequently, the knowledge gained during the initial training is transferred and can be applied to new experiments with different data types. Moreover, transfer learning allows for the development of experiments even with datasets containing limited samples. This is feasible because some pre-trained models have been trained on vast datasets from the web, encompassing millions of images across a diverse range of classes [37].

The following is a formal explanation of the transfer learning technique [38]:

A domain *D* is defined by two parts: a feature space X and a marginal probability distribution P(X), where $X=\{x_{1},...,x_{n}\}\in X$, $x_{i}$ is the *i*-*th* feature vector (instance), *n* is the number of feature vectors in *X*, X is the space of all possible feature vectors, and *X* is a particular learning sample. For a given domain *D*, a task *T* is defined by two parts: a label space *Y* and a predictive function f(·), which is learned from the feature vector and label pairs $\left\{x_{i},y_{i}\right\}$, where $x_{i}\in X$ and $y_{i}\in Y$.

Taking into account that a domain is expressed as D={X,P(X)} and a task is expressed as T={Y,f(·))}, a $D_{S}$ is defined as the source domain data, where $D_{S}=\left\{\left(x_{S_{1}},y_{S_{1}}\right),...,\left(x_{S_{n}},y_{S_{n}}\right)\right\}$, where $x_{S_{i}}\in X_{S}$ is the *i*-*th* data instance of $D_{S}$, and $y_{S_{i}}\in Y_{S}$ is the corresponding class label for $x_{S_{i}}$. In the same way, $D_{T}$ is defined as the target domain data, where $D_{T}=\left\{\left(x_{T_{1}},y_{T_{1}}\right),...,\left(x_{T_{n}},y_{T_{n}}\right)\right\}$, where $x_{T_{i}}\in X_{T}$ is the *i*-*th* data instance of $D_{T}$, and $y_{S_{i}}\in Y_{S}$ is the corresponding class label for $x_{T_{i}}$. Further, the source task is notated as $T_{S}$, the target task as $T_{T}$, and the source predictive function as $f_{T}\left(\cdot \right)$.

Then, given a source domain $D_{S}$ with a corresponding source task $T_{S}$ and a target domain $D_{T}$ with a corresponding task $T_{T}$, transfer learning is the process of improving the target predictive function $f_{T}\left(\cdot \right)$ by using the related information from $D_{S}$ and $T_{S}$, where $D_{S}\neq D_{T}$ or $T_{S}\neq T_{T}$.

### 2.3. The Network Architecture

The network architecture resulting from the different models implemented in this study was generated according to the following stages:

- Input Layer;
- Base Model;
- Global Average Pooling 2D;
- Dropout;
- Dense Layer;
- Output Layer.

**Figure 3:**
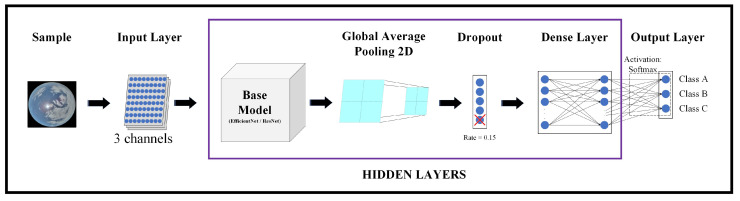
Representative diagram of the architecture.

A representative diagram of the architecture used in this survey can be seen in Figure 3.

The dataset was initially pre-processed by resizing the images to 200×200 pixels. As illustrated in the diagram depicting the utilized architecture, the Input Layer receives pixel values from the sample to be classified, comprising 200×200 pixels ×3 channels, where each channel represents a color in the RGB image.

Subsequently, the pre-processed data were fed into the chosen transfer learning model, serving as the backbone of the classifier. Each *Keras* application requires specific input pre-processing procedures, thus the pixel values were normalized based on the selected base model. For *EfficientNet* models (version 1 and version 2), the inputs were expected to be float tensors with pixel values ranging from 0 to 255. Conversely, for *ResNet* models, the RGB input images were converted to BGR format, followed by zero-centering each color channel with respect to the ImageNet dataset, without any scaling applied to the pixel values.

It is important to highlight that all base models underwent pre-training using the ImageNet database [39]. This entailed obtaining the weight values associated with the base model pre-trained using this database. ImageNet constitutes a vast ontology of images constructed upon the foundation of the WordNet structure [40].

Following this step, the Global Average Pooling 2D operation was employed, which computes the average value across spatial dimensions for multiple layers.

The Dropout Layer randomly deactivates input neural network units with a frequency determined by the rate parameter during training. This technique aids in mitigating overfitting by preventing reliance on specific neurons. Inputs that are not deactivated are proportionally scaled up by a factor of 1/(1 − rate), ensuring that the sum of all inputs remains consistent. In our architecture, the dropout rate was set to 15%.

Following the Dropout Layer, the Dense Layer, commonly known as the fully connected layer, comprises neurons connected to every neuron in the previous layer. Each neuron applies a specified activation function. In this study, we employed the *Softmax* activation function.

Finally, we have the Output Layer, which consists of as many neurons as there are classes in the dataset. Each output neuron applies the *Softmax* activation function. Thus, each neuron provides an estimate of the probability that the processed sample belongs to its corresponding class. In our study, with 3 classes of sky images, the architecture included 3 output neurons.

On the one hand, the *EfficientNet* models used as base models in the experiments of this research were as follows:

- *EfficientNet-B0*;
- *EfficientNet-B1*;
- *EfficientNet-B2*;
- *EfficientNet-B3*;
- *EfficientNet-B4*;
- *EfficientNet-B5*;
- *EfficientNet-B6*;
- *EfficientNet-B7*;
- *EfficientNetV2-B0*;
- *EfficientNetV2-B1*;
- *EfficientNetV2-B2*;
- *EfficientNetV2-B3*;
- *EfficientNetV2-S*;
- *EfficientNetV2-M*;
- *EfficientNetV2-L*.

It should be noted that the *EfficientNetV2-XL* model was not implemented in this study due to its unavailability in the *Keras* library at the time of writing. On the other hand, the *ResNet* models used as base models in the experiments were as follows:

- *ResNet-50*;
- *ResNet-101*.

## 3. Experimental Methodology

The objective of this experimentation was to assess the capability of the *EfficientNet* family and the specified *ResNet* models in distinguishing different cloudiness conditions in sky images. To achieve this, the models were evaluated using the network architecture outlined in Figure 3 by classifying the dataset described in Section 2.1.

In this section, a theoretical explanation is provided for the various methodologies employed to derive the results in this study.

### 3.1. k-Fold Cross-Validation Method

In the experiments of this survey, the *k*-fold cross-validation method was utilized to evaluate the performance of each classification model and to ensure the independence of the results from the partition between test and training data.

Cross-validation is a resampling technique employed to assess machine learning models with a limited dataset of samples. It involves iteratively computing the mean of evaluation metrics across various partitions of the dataset.

The entire database is utilized in each distribution of samples, encompassing both training and test samples. However, there are various approaches to distributing and employing the original dataset. In this context, two types of cross-validation can be discerned: exhaustive and non-exhaustive cross-validation.

- Exhaustive cross-validation methods involve learning and testing all possible ways to divide the original sample into a training and a validation set;
- Non-exhaustive cross-validation methods do not compute all possible ways of splitting the original sample.

Exhaustive cross-validation methods demand significant computational resources, especially considering the dataset dimensions in this study. In the case of *Leave-One-Out Cross-Validation (LOOCV)*, the model must be fitted repeatedly, equal to the number of samples, significantly increasing the computational time, particularly with three classes and 1500 samples per class. Hence, the cross-validation method utilized in these experiments was non-exhaustive, specifically, *k*-fold cross-validation (*k*-fold CV) as mentioned above.

In *k*-fold cross-validation, the dataset is randomly divided into *k* groups or folds of approximately equal size. Each fold is then used once as a test set while the model is trained on the remaining *k* − 1 folds. This process is repeated *k* times, where each time, a different group of samples is treated as a test set. As a result, *k* validations of the model are conducted, yielding the mean of the metrics used to evaluate the model.

In these experiments, the training and test datasets were divided into five groups (5-fold) to ensure each grouping contained an equal number of samples while having different samples. Subsequently, each model was trained using the training samples from each grouping. Following training, the test dataset was classified to obtain metrics from each model, facilitating evaluation based on these metric values. Finally, the results were computed as the mean of the metric values obtained across the different folds. It is worth noting that the training datasets differed slightly across the five groupings, as each model was generated from its own training dataset. This is significant as the training dataset heavily influences the adjustments of the model, despite all models being based on the same base model for each experiment. Essentially, *k*-fold cross-validation generates *k* different models, each trained on distinct sets of training samples but based on the same type of *Keras* model.

Since there were 4500 samples in total, 3600 training samples and 900 test samples were available in each *k*-fold, so that no sample from the test dataset was repeated in the five different groups.

During the model training process, the training dataset of each *k*-fold was further divided into two subsets: the training subset itself, utilized to train the model in each training cycle (*epoch*), and the validation subset. The validation split aids in iteratively enhancing the performance of the model by fine-tuning it after each epoch.

The test set provides the ultimate metrics of the model upon completing the training phase. Finally, the results were computed as the mean of the values of these metrics obtained across the various folds.

It is worth noting that the training dataset varied slightly across the five groupings, resulting in five distinct models from the same architecture. Each model was generated from its own training dataset, as the specifics of the training dataset influence the model adjustments. However, all models were based on the same base model type for each experiment.

### 3.2. Performance Metrics

To handle the diverse range of classification models, it becomes essential to utilize metrics or comparative frameworks that enable a qualitative analysis of the proposed models and facilitate the contrast of their outcomes. These metrics are applied to assess the efficacy of the algorithms in classification and, consequently, in identifying various types of cloudiness conditions in sky images.

The main performance metrics used to evaluate the models developed in this study were as follows: *Accuracy, Precision, Recall,* and *F1 Score*. The formulation of these metrics relies on the confusion matrix.

#### 3.2.1. Confusion Matrix

The confusion matrix is a technique employed to assess the accuracy of image classification algorithms. It operates under the assumption that the *ground truth* information possesses the following properties:

- Each image is labeled as belonging to a certain class so that there are *N* reference classes, ${\left\{R_{i}\right\}}_{i=1}^{N}$;
- Reference classes are mutually exclusive; that is to say, a certain image has no different classes (Equation (1)):
  (1) $$R_{i}\cap R_{j}=⌀,\nabla i\neq j$$

Suppose that each sample $R_{i}$ from a specific sky condition *S* to be evaluated is assigned by an algorithm as belonging to a certain class $C_{i}$, and, having *N* classes, the dataset $C_{i}$ determines only one specific sky condition to evaluate, meaning that two different sets have no element in common. Ultimately, there was no more than one sky condition of the three classes under study in each image in these experiments. This can be expressed mathematically as indicated in Equation (2).

(2) $$\cup_{i=1}^{N}C_{i}\in S\;\mathrm{and}\;C_{i}\cap C_{j}=⌀,\nabla i\neq j$$

In this theoretical framework, when establishing a binary classifier model, results can be categorized as positives (*p*) or negatives (*n*). Consequently, the prediction issue presents four potential outcomes from the classification process as follows:

- *TP* is true positive: a test result that correctly indicates the presence of a condition or characteristic;
- *TN* is true negative: a test result that correctly indicates the absence of a condition or characteristic;
- *FP* is false positive: a test result that wrongly indicates that a particular condition or attribute is present;
- *FN* is false negative: a test result that wrongly indicates that a particular condition or attribute is absent.

Based on the above, an experiment can be defined with *P* positive instances and *N* negative instances. The four potential outcomes can be represented in a 2×2 confusion matrix (Table 2).

From this confusion matrix, various metrics can be derived to evaluate the performance of different prediction models. As stated above, the performance of the classification algorithms was primarily evaluated using four metrics: *Accuracy*, *Precision*, *Recall*, and *F1 Score*.

#### 3.2.2. Accuracy

The *Accuracy* is defined as the fraction of correct predictions made by the classifier out of the total number of predictions. *Accuracy* can also be calculated in terms of positive and negative predictions as expressed in Equation (3):

(3) $$Accuracy=\frac{TP+TN}{TP+TN+FP+FN}$$

True positives and true negatives are the elements correctly classified by the model and they are on the main diagonal of the confusion matrix.

#### 3.2.3. Precision

The *Precision*, also called *Positive Predictive Value (PPV)*, is the fraction of test images classified as a specific class—as an example, class A—that are truly assigned to this class. *Precision* can be calculated as expressed in Equation (4):

(4) $$Precision=\frac{TP}{TP+FP}$$

#### 3.2.4. Recall

*Recall*, also known as *Sensitivity*, *Hit Rate*, or *True Positive Rate (TPR)*, is the fraction of test images from a class that are correctly identified to be assigned to this class. *Recall* can be calculated as expressed in Equation (5):

(5) $$Recall=\frac{TP}{TP+FN}$$

#### 3.2.5. F1 Score

The last two metrics can be used as parts of another metric taking *Precision* and *Recall* measures under the concept of harmonic mean. It could be interpreted as the *F1 Score*, which has its best value at 1 and worst value at 0. The *F1 Score* can be calculated as expressed in Equation (6):

(6) $$F1\;Score=2\cdot \left(\frac{Precision\cdot Recall}{Precision+Recall}\right)$$

The relative contribution of *Precision* and *Recall* are equal onto the *F1 Score*, and the harmonic mean is useful to find the best trade-off between the two quantities [41].

#### 3.2.6. Micro-Average, Macro-Average, and Weighted-Average

In contrast to situations involving multi-label classifiers, in multi-class classification, as is the case in this study where each observation was assigned a single label and Equation (1) holds true, the *F1 Score* needs to consider all classes. *Accuracy* is calculated similarly to binary classification. The Accuracy formula calculates the sum of true positive and true negative elements in the numerator and divides it by the sum of all entries in the confusion matrix in the denominator. True positives and true negatives represent the correctly classified elements by the model, located on the main diagonal of the confusion matrix. Moreover, the denominator includes all elements outside the main diagonal, representing those incorrectly classified by the model. However, the *F1 Score* requires a multi-class calculation of the *Precision* and *Recall* to be integrated into the harmonic mean, with various specifications available for these metrics. There are various approaches to implementation, including macro-averaging (*Macro-AVG*), micro-averaging (*Micro-AVG*), and weighted-averaging (*Weighted-AVG*).

#### 3.2.7. Micro-Average (Micro-AVG)

The aim was to evaluate all the units collectively, disregarding potential variations among classes. Initially, the aggregate counts of true positive (*TP*), false positive (*FP*), and false negative (*FN*) predictions across all classes were computed. Subsequently, performance metrics were derived based on these cumulative counts (Equations (7) and (8)).

(7) $$Micro\;AVG\;Precision=\frac{\sum_{i=1}^{N}TP_{C_{i}}}{\sum_{i=1}^{N}TP_{C_{i}}+\sum_{i=1}^{N}FP_{C_{i}}}$$

(8) $$Micro\;AVG\;Recall=\frac{\sum_{i=1}^{N}TP_{C_{i}}}{\sum_{i=1}^{N}TP_{C_{i}}+\sum_{i=1}^{N}FN_{C_{i}}}$$

Given that each false positive (*FP*) for one class equated to a false negative (*FN*) for another class, the count of *FN* and *FP* yielded identical values. Consequently, both *Micro-AVG Precision* and *Micro-AVG Recall* metrics produced the same result.

Taking into account the definition in Equation (6), it can be deduced that the *Micro-AVG F1 Score* also gives the same result (the harmonic mean of two equal values is just the value), and taking a look at the formulas, it is the same formula to calculate the overall *Accuracy*. This equality is expressed in Equation (9).

(9) MicroAVGF1Score=MicroAVGPrecision=MicroAVGRecall=Accuracy

#### 3.2.8. Macro-Average (Macro-AVG)

In this case, *Precision* was computed by averaging the *Precision* scores for each predicted class, while *Recall* was determined by averaging the *Recall* scores for each actual class.

*Precision* and *Recall* can be calculated individually for each class, resulting in multiple metrics depending on the number of classes. These metrics were computed by treating each class as the positive instance while considering all other classes as a single negative instance. When transitioning from one class to another, the quantities were recalculated, and the labels for the confusion matrix tiles were adjusted accordingly. This approach allowed the derivation of these metrics for each class. Equations (10) and (11) show how *Precision* and *Recall* were, respectively, computed for the class $C_{i}$:

(10) $$Precision_{C_{i}}=\frac{TP_{C_{i}}}{TP_{C_{i}}+FP_{C_{i}}}$$

(11) $$Recall_{C_{i}}=\frac{TP_{C_{i}}}{TP_{C_{i}}+FN_{C_{i}}}$$

Macro-Average *Precision* and *Recall* were simply computed as the arithmetic mean of the metrics for single classes. So, these metrics can be obtained as is shown in Equation (12) and Equation (13), respectively:

(12) $$Macro\;AVG\;Precision=\frac{\sum_{i=1}^{N}Precision_{C_{i}}}{N}$$

(13) $$Macro\;AVG\;Recall=\frac{\sum_{i=1}^{N}Recall_{C_{i}}}{N}$$

Finally, the *Macro AVG F1 Score* is the harmonic mean of *Macro AVG Precision* and *Macro AVG Recall* (Equation (14)):

(14) $$Macro\;AVG\;F1\;Score=2\cdot \left(\frac{Macro\;AVG\;Precision\cdot Macro\;AVG\;Recall}{Macro\;AVG\;Precision+Macro\;AVG\;Recall}\right)$$

In this approach, there is no consideration of the size of the sample set for each class, as classes of different sizes carry the same weight in the numerator. This implies that the effect of classes with a larger number of samples has the same importance as that of classes with a smaller number of samples. The resulting metric evaluates the algorithm from the class point of view: high *Macro AVG F1 Score* values indicate that the algorithm performs well across all classes, while low *Macro AVG F1 Score* values indicate poorly predicted classes.

#### 3.2.9. Weighted-Average (Weighted-AVG)

This approach considers the class balance by assigning weights to each class based on its representation in the dataset. Performance metrics are then computed as a weighted-average of these metrics across individual classes. Weighted-averaging is particularly useful in unbalanced datasets, where greater importance is assigned to classes with more samples.

However, given the utilization of a balanced dataset in this study, where each class carries equal significance, it became imperative to discern any potential bias in class classification relative to others. Consequently, the *Macro-Average* approach was adopted to derive the results outlined in Section 4.

## 4. Results and Discussion

This section outlines the outcomes derived from the classification experiments conducted with the implemented models. Using these results as a basis, various aspects of the comparison are subsequently discussed.

In all experiments, each training session utilized the adaptive optimization algorithm *Adam* and employed the *Sparse Categorical Cross-Entropy* as the loss function. Additionally, each training session was conducted with a maximum of 100 epochs, a batch size of 16 samples, and a validation percentage of 0.2, where the *Early Stopping* mechanism was incorporated to halt training if there was no improvement for 20 epochs, with *Accuracy* as the monitored metric.

Table 3 and Table 4 present the maximum number of epochs for training the models along with the parameter count for each model. The first column provides row identifiers for clarity, while the second column lists the names of the base models. The third column denotes the maximum number of epochs, corresponding to the *k*-fold with the highest number of epochs. The fourth column displays the total number of parameters in the entire network, and the last column specifies the count of trainable parameters.

As can be seen in Table 3, none of the models using residual neural networks reached the maximum number of epochs. In contrast, as can be seen from Table 4, in experiments utilizing the base models *EfficientNet-B0*, *EfficientNet-B1*, *EfficientNet-B4*, and *EfficientNetV2-B0*, the number of epochs reached aligns with the maximum epoch limit set by the epoch hyperparameter.

Moreover, it also can be observed that the model utilizing the residual architecture *ResNet-101*, comprising 42,664,323 parameters, was surpassed in terms of the total parameter count only by models employing the *EfficientNet-B7*, *EfficientNetV2-M*, and *EfficientNetV2-L* architectures, with the latter model having the highest number: 117,750,691 parameters. This was despite the fact that the model implementing the *EfficientNet-B7* architecture possessed the highest number of trainable parameters, totaling 7683 parameters.

Nevertheless, as can be seen below, neither the greater number of trainable parameters, total parameters, nor the greater number of epochs consumed during the training proved to be decisive in determining the model that exhibited the best performance in the conducted experiments.

This suggests that, by increasing this threshold, there is potential to enhance results with these base models. A higher number of epochs could lead to further refinement in the learning process of the model, thereby improving its overall performance.

Nevertheless, additional experiments were conducted with a maximum limit of 200 epochs. Within these experiments, both the *EfficientNet-B0* and *EfficientNet-B1* base models were tested. However, only *EfficientNet-B0* surpassed 100 training epochs, reaching 112 epochs in one of its folds. Despite these prolonged training durations, none of the experiments showcased superior performance compared to the results presented in this paper. It should be noted that, although the applied *k*-fold cross-validation method ensures the independence of partitioning results between test and training data, a random component exists in the system configuration depending on the randomly chosen data during the training phase, which can slightly influence their performance.

On the other hand, as the 5-fold cross-validation method was employed, five models were generated for each base model. This signifies that, for each metric utilized, there were five values obtained from the test phase of each corresponding fold. Consequently, the mean value and standard deviation of these 5-fold results were utilized to assess the performance of each architecture.

Table 5 and Table 6 show the metrics obtained using the *ResNet* and *EfficientNet* architectures, respectively—*Accuracy*, *Precision*, *Recall*, and *F1 Score*—depending on the base model integrated in each model. Similar to the preceding tables, the first column comprises row identifiers for clarity, while the second column includes the names of the base models. The subsequent columns present the values of these metrics.

Given the favorable outcomes observed across all experiments in terms of *Precision*, *Recall*, and therefore, *F1 Score* metrics, with values between 97.40% and 98.20%, the primary focus for comparing the classification capabilities among the various models was placed on *Accuracy*. This decision stems from the comprehensive evaluation of *Precision*, *Recall*, and *F1 Score* metrics, which collectively indicated satisfactory performance across different models.

In other words, the *Precision* and *Recall* results obtained in each experiment were very close to each other, often with identical values, and consistently high, accompanied by negligible standard deviations. This suggests that there was no significant bias favoring certain classes over others, thus affirming the reliability of *Accuracy* as the primary metric for comparison. By prioritizing *Accuracy* as the main metric, we intended to provide a comprehensive assessment of the overall classification efficacy of each model, considering its ability to correctly classify instances across all classes.

Continuing with the analysis of the results presented in both Table 5 and Table 6, it can be observed that the models utilized in this study, whether based on the *ResNet* or *EfficientNet* architectures, demonstrated notably high levels of *Accuracy*. In particular, the lowest performing model was the one employing the *EfficientNet-B7* architecture, which achieved an *Accuracy* of 97.31%, with a standard deviation of 0.35. Despite having the highest number of trainable parameters and the second highest number of total parameters, it was the sole model within the *EfficientNet* family that failed to surpass the performance of the models utilizing residual architectures in this study.

The models utilizing residual neural networks in this survey achieved a slightly lower performance compared to those employing *EfficientNet* architectures, with the exception of the model highlighted in the preceding paragraph. This is clearly reflected in the differences in the results of the *Accuracy* metric. Of the models implementing *EfficientNet* architectures, *EfficientNet-B7* was followed in performance by the one using the *EfficientNetV2-S* architecture. While the latter achieved an *Accuracy* of 97.60% with a standard deviation of 0.54, the *ResNet-50* model, from the residual neural network family, offered a slightly lower *Accuracy* of 97.56% with a standard deviation of 0.42.

Regarding the outcomes derived from version 1 and version 2 of the *EfficientNet* architectures, they did not establish a clear distinction in terms of performance disparity between the two versions. Consequently, the results did not suggest that models from one version outperformed those from the other. Instead, models from both versions exhibited comparable performance, with their results hovering around similar values.

Nevertheless, the most promising outcomes were attained with *EfficientNetV2-B1* and *EfficientNetV2-B2* as the base models. Remarkably, both architectures yielded identical results, boasting a mean *Accuracy* of 98.09% with a standard deviation of 0.34.

Following closely, the model demonstrating the third-best performance utilizes the *EfficientNet-B1* architecture (version 1). This model delivered a mean *Accuracy* of 98.07%, accompanied by a standard deviation of 0.32.

In this comparative analysis, it is evident that all the base models utilized in this study, derived from both the *EfficientNet* and *ResNet* families, exhibited mean values of *Accuracy*, *Precision*, *Recall*, and *F1 Score* exceeding 97% with a standard deviation below 1. Consequently, all of these models warrant consideration for similar applications aimed at identifying cloud conditions. Particularly noteworthy are *EfficientNetV2-B1* and *EfficientNetV2-B2*, which emerged as the top-performing base models and thus merit special attention. Nonetheless, *EfficientNet-B1*, *EfficientNet-B3*, *EfficientNetV2-B1*, *EfficientNetV2-B2*, and *EfficientNetV2-B3* also deserve consideration, as they consistently delivered values exceeding 98% across all performance metrics.

Furthermore, to highlight the significance of this research within the current state of the art, it is essential to contextualize the differences between the results obtained in this study and those of other investigations that also utilized *EfficientNet*, *ResNet* models, as well as others CNN-based architectures for classifying sky images. Below are several studies that developed models for the classification of sky images.

For instance, the research conducted by Joby M. Prince Czarnecki et al. [10] employed two *ResNet* architectures and achieved their best result with the *ResNet-34* model. Similarly, the study by Muhammad Umair and Manzoor Ahmed Hashmani [19] utilized *GoogLeNet*, *ResNet-50*, and *EfficientNet-B0* architectures, with their best performance being obtained using the *EfficientNet-B0* model. As mentioned in the introduction, Emmanuel Kwabena Gyasi and Purushotham Swarnalatha [9] obtained their best outcome using the *Cloud-MobiNet* architecture in their survey. Lastly, the research carried out by Mehmet Guzel, Muruvvet Kalkan, et al. [12], where they used *EfficientNetV2-L* and *ResNetV2-152* among others, obtained the best result with a *Xception* model.

By comparing the findings of this study with those of other investigations, the advancements and unique contributions to the field are highlighted. For this reason, Table 7 presents the best results from the above-mentioned studies in contrast with the best result obtained in this work.

It is important to note that the information provided in Table 7 aims to provide insight into the performance of various architectures utilized in different studies. It should be emphasized that the datasets employed in each of these studies differ from the one used in this study; hence, this table includes both the number of samples and the number of classes used in each study. Additionally, although these studies address diverse forms of cloud cover, there is variation in both the type and format of the imagery. While not the focus of this investigation, for a rigorous comparison of these models, employing exactly the same dataset would be necessary.

## 5. Conclusions

This study presents an extensive exploration of the application of models from the *EfficientNet* family and residual neural network architectures for automating the identification of cloud conditions from sky images.

The findings of this investigation underscore the suitability of these models as base models for the architecture utilized in this study. Notably, they highlight the potential of employing transfer learning techniques within convolutional neural network frameworks, particularly in their ability to deliver high-performance outcomes. The comparison encompasses 17 distinct models sourced from the models available in the *Keras* library, including both versions of the *EfficientNet* architectures and the *ResNet-50* and *ResNet-101* models from residual neural networks.

Upon analyzing the *Accuracy* results and conducting a comprehensive comparative assessment, it was concluded that while there were subtle distinctions in the classification performance across these models, certain models exhibited a propensity for superior performance in this application domain. Nonetheless, the *Precision*, *Recall*, and *F1 Score* metrics consistently indicate that all models provided unbiased classification across the considered classes, taking into account that a balanced dataset was used.

Furthermore, this study underscores the potential integration of certain implemented models into practical systems designed for cloud condition identification from sky images captured by hemispherical sky cameras. One potential application area highlighted is in forecasting renewable energy generation in photovoltaic infrastructure, where these models demonstrated promising performance in image classification.

Among the array of models compared, standout performers include those implementing the base models *EfficientNetV2-B1* and *EfficientNetV2-B2*. These models exhibited superior performance, particularly in terms of the *Accuracy* and *F1 Score* metrics, indicating minimal bias in their classification outputs.

Moving forward, subsequent lines of inquiry could explore the improvement of the most favored models through techniques such as data augmentation and architectural modifications. Additionally, there is potential for investigating alternative architectures, such as Transformers and their corresponding attention layers, to further enhance performance in this domain.

## Figures and Tables

**Table 1:** Dataset of the selected samples.

Class	Number of Samples	Color Model	Format	Aspect Ratio (pixels)
Clear	1500	RGB	PNG	400×400
Cloudy	1500	RGB	PNG	400×400
Partly Cloudy	1500	RGB	PNG	400×400

**Table 2:** Confusion matrix (2×2).

		PREDICTION	
		**Positive Prediction**	**Negative Prediction**
**GROUND-TRUTH** **CONDITION**	**Positive Condition**	True Positives (TP)	False Negatives (FN)
	**Negative Condition**	False Positives (FP)	True Negatives (TN)

**Table 3:** Number of epochs (maximum) and parameters in the implemented models (ResNet).

ID	Base Model	No. Epochs (Maximum)	Parameters (Weights + Biases)	
			**Total**	**Trainable**
1	ResNet-50	74	23,593,859	6147
2	ResNet-101	38	42,664,323	6147

**Table 4:** Number of epochs (maximum) and parameters in the implemented models (EfficientNet).

ID	Base Model	No. Epochs (Maximum)	Parameters (Weights + Biases)	
			**Total**	**Trainable**
1	EfficientNet-B0	100	4,053,414	3843
2	EfficientNet-B1	100	6,579,082	3843
3	EfficientNet-B2	95	7,772,796	4227
4	EfficientNet-B3	98	10,788,146	4611
5	EfficientNet-B4	100	17,679,202	5379
6	EfficientNet-B5	62	28,519,674	6147
7	EfficientNet-B6	78	40,967,058	6915
8	EfficientNet-B7	63	64,105,370	7683
9	EfficientNetV2-B0	100	5,923,155	3843
10	EfficientNetV2-B1	91	6,934,967	3843
11	EfficientNetV2-B2	67	8,773,601	4227
12	EfficientNetV2-B3	68	12,935,233	4611
13	EfficientNetV2-S	81	20,335,203	3843
14	EfficientNetV2-M	86	53,154,231	3843
15	EfficientNetV2-L	77	117,750,691	3843

**Table 5:** Values of the metrics in the implemented models (ResNet).

ID	Base Model	*Accuracy*	*Precision*	*Recall*	*F1 Score*
		**Mean (%) (Standard Deviation)**			
1	ResNet-50	97.56 (0.42)	97.40 (0.55)	97.40 (0.55)	97.40 (0.55)
2	ResNet-101	97.49 (0.58)	97.40 (0.89)	97.40 (0.89)	97.40 (0.89)

**Table 6:** Values of the metrics in the implemented models (EfficientNet).

ID	Base Model	*Accuracy*	*Precision*	*Recall*	*F1 Score*
		**Mean (%) (Standard Deviation)**			
1	EfficientNet-B0	97.67 (0.46)	97.60 (0.55)	97.60 (0.55)	97.60 (0.55)
2	EfficientNet-B1	98.07 (0.32)	98.20 (0.45)	98.00 (0.00)	98.20 (0.45)
3	EfficientNet-B2	97.82 (0.38)	97.80 (0.45)	97.80 (0.45)	97.80 (0.45)
4	EfficientNet-B3	98.02 (0.42)	98.20 (0.45)	98.20 (0.45)	98.20 (0.45)
5	EfficientNet-B4	97.98 (0.24)	98.00 (0.00)	98.00 (0.00)	98.00 (0.00)
6	EfficientNet-B5	97.87 (0.28)	98.00 (0.00)	98.00 (0.00)	98.00 (0.00)
7	EfficientNet-B6	97.69 (0.45)	97.60 (0.55)	97.60 (0.55)	97.60 (0.55)
8	EfficientNet-B7	97.31 (0.35)	97.40 (0.55)	97.40 (0.55)	97.40 (0.55)
9	EfficientNetV2-B0	97.76 (0.28)	97.80 (0.45)	97.80 (0.45)	97.80 (0.45)
10	EfficientNetV2-B1	**98.09 (0.34) ^1^**	98.00 (0.00)	98.00 (0.00)	98.00 (0.00)
11	EfficientNetV2-B2	**98.09 (0.34) ^1^**	98.00 (0.00)	98.00 (0.00)	98.00 (0.00)
12	EfficientNetV2-B3	98.00 (0.25)	98.00 (0.00)	98.00 (0.00)	98.00 (0.00)
13	EfficientNetV2-S	97.60 (0.54)	97.60 (0.55)	97.60 (0.55)	97.60 (0.55)
14	EfficientNetV2-M	97.76 (0.52)	97.80 (0.45)	97.80 (0.45)	97.80 (0.45)
15	EfficientNetV2-L	97.89 (0.39)	98.00 (0.71)	97.80 (0.84)	97.80 (0.84)

^1^ Best outcomes.

**Table 7:** Comparison of best results from previous studies and the current work.

Research	Underlying Architecture	Overall Accuracy (%)	No. Samples	No. Classes
*Czarnecki et al. (2021)*	EfficientNet-B0	87.2	600 ^1^	6
*Umair et al. (2022)*	ResNet-34	90	13,000	2
*Gyasi et al. (2023)*	MobileNet	97.45	2543 ^2^	11 *
*Guzel et al. (2024)*	Xception	97.66	2543 ^3^	11 *
Our survey	EfficientNetV2-B1/B2	98.09	4500	3

^1^ The initial dataset of 600 samples was augmented using data augmentation methods to produce 9600 samples. ^2^ Out of the 2543 images, data augmentation was applied to 2433 samples (training samples). ^3^ After applying data augmentation, the image set with 2543 images increased more than tenfold. * One of the classes used in these studies does not correspond to a cloud condition but rather to the vapor trails of airplanes.

## Data Availability

The data presented in this study are available on request from the corresponding author.

## Abbreviations

The following abbreviations are used in this manuscript:

ANN	Artificial Neural Network
API	Application Programming Interface
ARM	Atmospheric Radiation Measurement
CMV	Cloud Motion Vectors
CNN	Convolutional Neural Network
DL	Deep Learning
DNI	Direct Normal Irradiance
DNN	Deep Neural Network
FCN	Fully Convolutional Network
FN	False Negative
FP	False Positive
GRU	Gated Recurrent Unit
HYTA	Hybrid Thresholding Algorithm
LOOCV	Leave-One-Out Cross-Validation
LSTM	Long Short-Term Memory
ML	Machine Learning
PPV	Positive Predictive Value
RGB	Red Green and Blue
RNN	Recurrent Neural Network
SGP	Southern Great Plains
SIRTA	Site Instrumental de Recherche par Télédétection Atmosphérique
SKIPP’D	SKy Images and Photovoltaic Power Generation Dataset
SWIMCAT	Singapore Whole-sky IMaging CATegories
TL	Transfer Learning
TN	True Negative
TP	True Positive
TPR	True Positive Rate
TSI	Total Sky-Imager
U-Net	U-shaped Network
